# Thirty per cent contrast in secondary-electron imaging by scanning field-emission microscopy

**DOI:** 10.1098/rspa.2016.0475

**Published:** 2016-11

**Authors:** D. A. Zanin, L. G. De Pietro, Q. Peter, A. Kostanyan, H. Cabrera, A. Vindigni, Th. Bähler, D. Pescia, U. Ramsperger

**Affiliations:** Laboratory for Solid State Physics, ETH Zurich, 8093 Zurich, Switzerland

**Keywords:** scanning tunnelling microscopy, secondary-electron imaging, scanning field-electron microscopy, scanning field-emission microscopy

## Abstract

We perform scanning tunnelling microscopy (STM) in a regime where primary electrons are field-emitted from the tip and excite secondary electrons out of the target—the scanning field-emission microscopy regime (SFM). In the SFM mode, a secondary-electron contrast as high as 30% is observed when imaging a monoatomic step between a clean W(110)- and an Fe-covered W(110)-terrace. This is a figure of contrast comparable to STM. The apparent width of the monoatomic step attains the 1 *nm* mark, i.e. it is only marginally worse than the corresponding width observed in STM. The origin of the unexpected strong contrast in SFM is the material dependence of the secondary-electron yield and not the dependence of the transported current on the tip–target distance, typical of STM: accordingly, we expect that a technology combining STM and SFM will highlight complementary aspects of a surface while simultaneously making electrons, selected with nanometre spatial precision, available to a macroscopic environment for further processing.

## 1. Introduction

**Figure 1.**
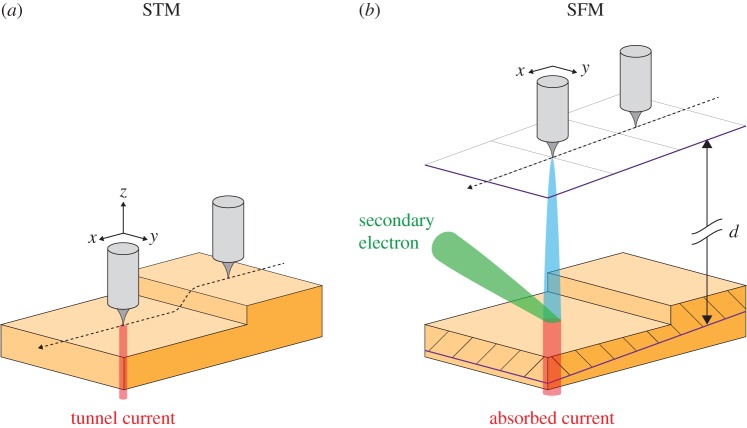
(*a*) STM imaging of a surface (in the present case, two ‘yellow’ terraces separated by a monoatomic step) is performed by scanning the tip (grey) along a horizontal *xy*-coordinate system and displacing the tip vertically along *z* so that the tunnel current (red) remains constant during the scan. (*b*) In SFM, the tip is translated along an *xy*-plane (indicated by the grid) parallel to the imaged surface area at an average distance *d*. Note that *d* is typically one order of magnitude larger than in STM. The tip is at a negative voltage of few −10 V with respect to the target. The field-emitted electrons build a primary beam (blue). The absorbed current (red) and the secondary-electron current escaping the junction (green) are measured during the scan.

In scanning tunnelling microscopy (STM), the distance between the tip and the target is in the subnanometre range. Accordingly, electrons can be exchanged by direct quantum mechanical tunnelling (figure 1*a*) between the outermost tip-atom and the top-most target-atom. The exponential dependence of the tunnelling probability on the tip-target distance produces the two distinct features that make STM almost unique: a subnanometre horizontal spatial resolution [1] and a strong image ‘contrast’. If one defines the contrast between the STM images of objects *a* and *b* as the difference between the tunnel currents arising from *a* and *b* divided by their sum,^1^ then [3], eqn. 8 the tunnel current in STM has a figure of contrast of few 10% (i.e. close to 100%, the maximum figure of contrast attainable) between terraces separated by a monoatomic step.

If the tip is retracted to a few nanometres or tens of nanometres (figure 1*b*), the instrument is operating in the Field Emission STM regime [4] (or ‘topografiner’ regime), described by Young [5,6] almost 10 years *in advance* of the invention of STM: some electrons (blue in figure 1*b*) are actually field-emitted through the tunnelling barrier surrounding the tip apex into the region of space, residing between the tip and the target. Upon striking the target, they are not only partly absorbed (red in figure 1*b*), but also generate ‘secondary electrons’:^2^ those (green in figure 1*b*) that escape the tip–target junction and reach the macroscopic environment surrounding it can be detected, for example, to produce a ‘secondary-electron image’ of the target itself—the proximity between tip and target acting to spatially localize the primary beam (blue in figure 1*b*). The STM turns, by the ‘simple’ operation of retracting the tip, into a lensless low-energy scanning electron microscope with the possibility of secondary-electron imaging [4,7–13].

In this article, we demonstrate an unexpectedly strong image contrast in secondary-electron imaging scanning field-emission microscopy (SFM). We find on one side that both the contrast and lateral resolution deteriorate rapidly with tip–target distance when imaging is based just on the recording of the transported current (red and/or blue in figure 1*b*). However, when secondary electrons (green in figure 1*b*) are used for imaging, the figures of contrast and horizontal resolution typical of STM are, surprisingly, (almost) recovered. Specifically, we have imaged a submonolayer Fe film grown, by Molecular Beam Epitaxy, on top of a W(110)-surface [14,15]. We observe that the secondary-electron contrast, when going across a monoatomic thick step dividing an Fe-terrace from the W-surrounding, can be as high as 30%. The width of the step is measured to reach the 1 *nm* mark. A strong secondary-electron contrast is also observed when going from a side of a surface consisting of Fe to a side of the *same* surface consisting of W, but no contrast is observed on a terrace consisting of the same atoms. These observations point to the element specificity of the strong secondary-electron contrast so that a technology combining STM and SFM should provide a table-top instrument for elemental fingerprinting of materials at the nanoscale [16].

## 2. Experiment

The images presented in this report refer to the 110-surface of a W-single crystal and submonolayers of Fe film grown on top of it by Molecular Beam Epitaxy. The experimental details related to the STM instrument and its use in the SFM regime, the tip and sample preparation, and secondary-electron detection are discussed elsewhere [12,13]. All images are taken at room temperature. The most striking technical feature of the present work—uncommon to any other previous work—is the capability of performing both STM and SFM imaging of the very same region of the target. Typically, in a first step, the tip is approached to subnanometre distances from the surface for STM imaging, which is performed by scanning the tip along a preset *xy*-plane while recording the displacement along *z* that the tip has to make in order to keep the tunnel current constant (figure 1*a*). After direct tunnelling imaging, the constant current feedback loop is turned off and the tip is retracted to a distance *d* from the surface, where *d* amounts to few nanometres to few tens of nanometres (figure 1*b*)—i.e. at least one order of magnitude to two orders of magnitude larger than in STM imaging. The tip is biased with a negative voltage *V* of the order of a few −10 V with respect to the target, so that a primary, field-emitted electron beam (blue in figure 1*b*) is effectively travelling towards the target. Note that, after STM imaging, the software interpolates the encoded tip deviations as a function of the *xy*-position by means of a mathematical plane.^3^ This defines a planar coordinate system (the grid in figure 1*b*) *parallel* to the previously imaged area, along which the tip is scanned during SFM imaging, which is therefore performed at a ‘constant average distance *d*’ between tip-apex and target. Two quantities are measured during SFM: the local current absorbed by the target (red in figure 1*b*), leading to absorbed current imaging SFM, and the secondary-electron current escaping the tunnel junction (green in figure 1*b*) and arriving to the detector, leading to secondary-electron imaging SFM. All images typically consist of 256×256 pixels. The scan rate for one line is 1 s in STM and 0.5 *s* in SFM. Imaging is performed at in ultra-high vacuum conditions (typically about 10^−10^ mbar). We have not yet tried SFM operation in worse vacuum conditions.

## 3. Results

**Figure 2.**
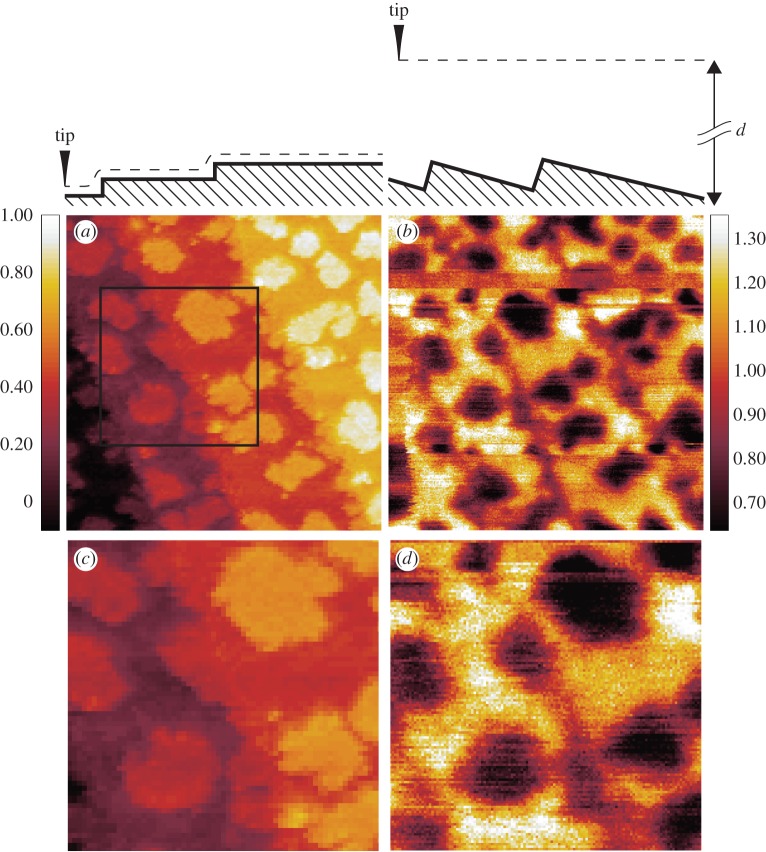
(*a*–*d*) STM and SFM images. For more details, see the caption of figure 3.

**Figure 3.**
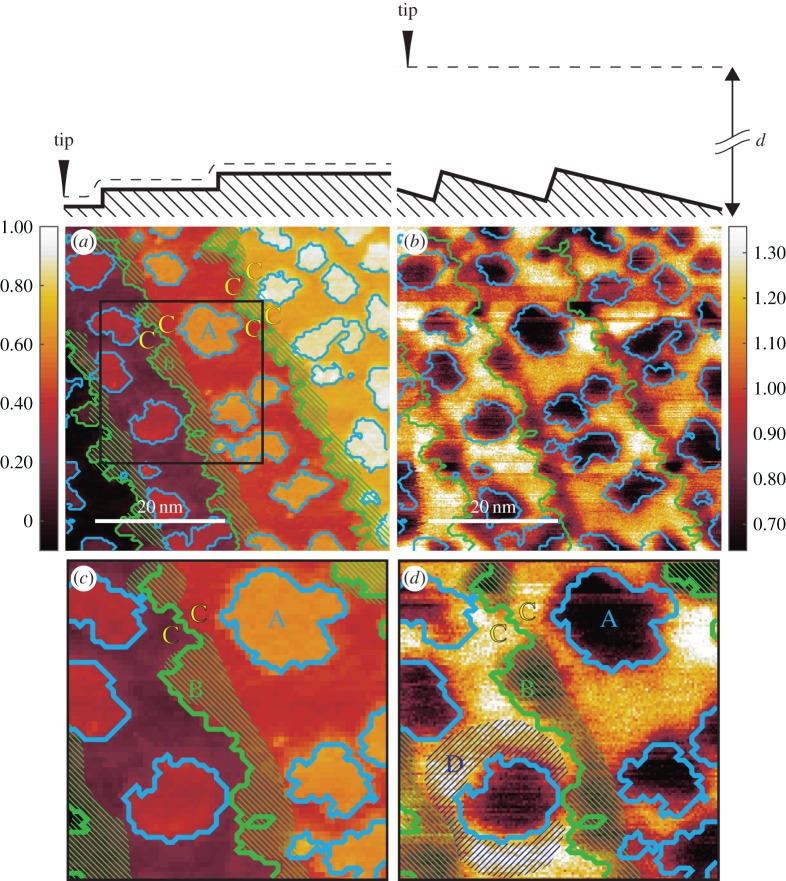
The same images of figure 2 with guiding marks. (*a*) STM image of ≈0.34 atomic layer of Fe grown epitaxially on top of a W(110) surface. A colour code (vertical bar) is used to render the tip displacement in *nm*. The surface profile seen by the tip apex while moving from left to right is sketched at the top of the image. The letters ‘A’, ‘B’ and ‘C’ and the dashed areas mark special domains of the surface: their meaning is discussed in the main text. The white horizontal line indicates both vertical and horizontal spatial dimensions. Continuous green and blue lines join those sites at which a one-step thick tip displacement is recorded, forming a kind of image skeleton which is then superimposed onto the following images. The area within the square window is enlarged in (*c*). (*b*) The same area as in figure 2*a* imaged with secondary-electron SFM. A colour code (vertical bar) is used to render the ratio of the local secondary-electron intensity divided by its line average. The white horizontal line indicates both vertical and horizontal spatial dimensions. The skeleton obtained in (*a*) is superimposed. (*c*) The area within the square window marked in the STM figure 2*a*. The A, B and C domains are also marked. (*d*) The same area as (*c*) from the corresponding secondary-electron image SFM (*b*). Note a technical detail: the entire type ‘A’ domains or the entire surroundings, see the blue-dashed region marked with D, are rendered (to within experimental noise) *uniformly* in the secondary-electron SFM image. This means that we do not observe any sizeable ‘edge enhancement’ of the secondary-electron production [10], nor are secondary electrons shadowed [10] by ‘obstacles’ (electric fields, protruding surface atoms, tip, etc.) on their way to the entrance of the detector.

Figure 2 shows the main images we would like to discuss in this paper. The same images are also reproduced as figure 3, but contain guiding marks, which we regard as very instructive. Accordingly, in the following, we refer to the images with guiding marks, but, in particular, when referring to the spatial resolution, we invite the reader to directly cross-check with the images without guiding marks.

### (a) Scanning tunnelling microscopy imaging

The STM image figure 3*a* (and the zoomed area in figure 3*c*) define the system we are going to study by SFM. Figure 3*a* shows an area of a W(110)-surface covered with about 0.34 monolayers of Fe. The colour code used to render the tip displacement (in nanometre) is given as a vertical bar. Equi-tip-displacement domains are rendered with the same colour. A ‘skeleton’ (continuous green and blue lines), indicating the sites at which the tip undergoes a vertical displacement corresponding to one atomic step (about 0.2 *nm*), has been constructed by applying a suitable algorithm. Accordingly, the image in figure 3*a* consists of four terraces, the deepest one being on the left-hand side (dark). The tip-displacement across this terrace is set to be the origin of the tip-displacement scale. A schematic profile of the surface, ‘seen’ by the tip while moving from left to right, is sketched by the thick line drawn on top of the image. On top of the terraces, one observes one monolayer of thick domains of variable size (in the following called type ‘A’ domains), enclosed within a blue contour. As the ‘clean’ W(110) surface consists of flat, wide terraces separated by monoatomic steps [15] (see also figure 9 in appendix C), one associates the ‘A’ domains with Fe-deposits and the surroundings with the W(110) surface. The relative area covered by the ‘A’ domains leads to the coverage of 0.34 monolayer Fe mentioned above. Note, however, the irregular geometry of the monoatomic steps running approximately along the diagonal of the image (continuous green lines) separating successive terraces: the irregularity is specific to the Fe-covered surface, as the steps of the ‘clean’ W(110) surface are almost straight on this spatial scale (see [15] and figure 9), and is therefore suggestive of *some* amount of Fe residing on the right-hand side of the steps as well. Note that these putative Fe-atoms are at the same level of displacement as the W-atoms further right along the same terrace and do not appear as a separate feature in the STM image figure 3*a*,*c*. Our marking further domains of figure 3*a*,*c* with a different set of letters (the ‘B’ domains, dashed in green, the ‘C’ domains) is a consequence of secondary-electron SFM imaging and will be explained in the next section as one of the central results of this work. We point out that STM image in figure 3*a*,*c* can also be read according to a proximity rule. While moving from left to right, the tip encounters successive domains that are positioned closer to its apex. The feedback-loop registers an increase of the tunnelling current and moves the tip farther away from the surface—a positive displacement which is rendered by a brighter colour. Therefore, in terms of this proximity rule of thumb ‘brighter colour’ in figure 3*a*,*c* also means ‘larger tunnel current’.

### (b) Secondary-electron scanning field-emission microscopy imaging

The image in figure 3*b* (and the zoomed area in figure 3*d*) is a secondary-electron image SFM of the same area of figure 3*a*, respectively, figure 3*c*. Note that the primary emission current in a field emission experiment is subject to fluctuations during the data taking, and with it the secondary electron current. To eliminate these fluctuations at least partially, we always divide, at each pixel, the secondary-electron current by the absorbed current. In addition, the local value is further divided by the line average, to obtain the image contrast explicitly. The colour code used to render the normalized secondary-electron current is given in the vertical bar, darker tones corresponding to smaller relative currents. The skeleton obtained in figure 3*a* is superimposed as continuous lines—as a guide to ‘read’ the image—in such a way that all prominent features are globally best aligned (locally, unavoidable drift produces some misalignment, compare figure 3*c*,*d* for details on this issue). Figure 3*b*,*d* contains two striking experimental facts that define the key observations of this article.

**Figure 4.**
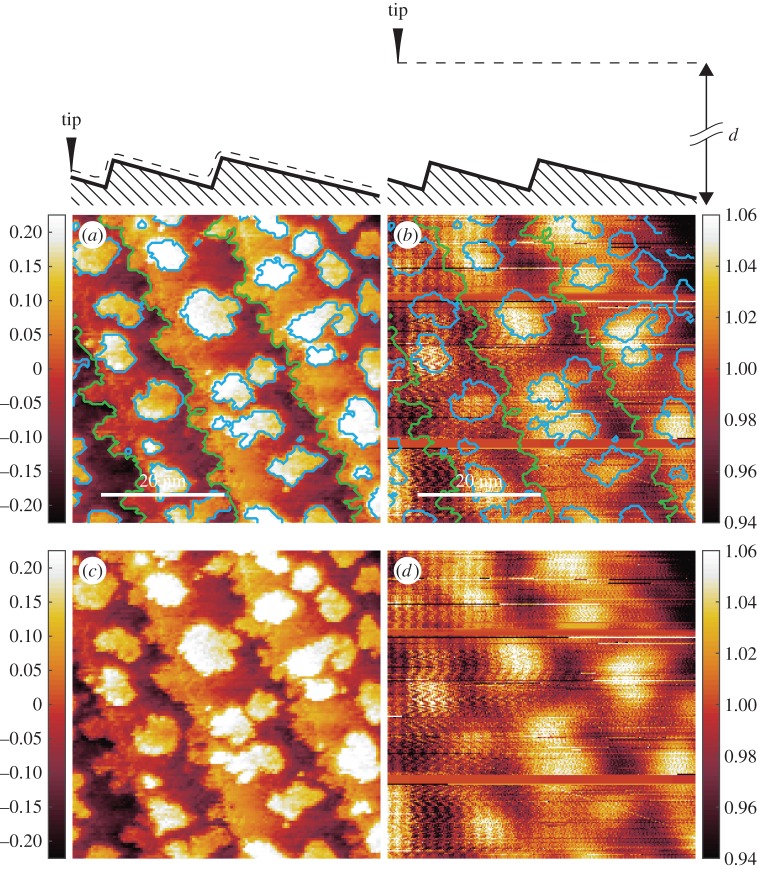
(*a*) The STM image figure 2*a* is rendered after subtraction of a plane parallel to the scanned area. A colour code (vertical bar) is used to render the tip displacement, in nanometres. The surface profile faced by the tip apex while moving from left to right is sketched at the top of the image. The colour change within a terrace is due to the tip needing to adjust to the sawtooth profile. The white horizontal line indicates both vertical and horizontal spatial dimensions. The skeleton obtained in figure 2*a* is superimposed. (*b*) The same area as in (*a*), imaged with absorbed current (*V* =−29.6 *V*, *d*=7±1 *nm*, average current *I*=400 *nA*). A colour code (vertical bar) is used to render the normalized absorbed current (i.e. the locally absorbed current divided by its line average). The white horizontal line indicates both vertical and horizontal spatial dimensions. On top a sketch of the surface profile is faced by the tip while scanning. The skeleton obtained in figure 2*a* is superimposed. (*c*,*d*) Images (*a*,*b*) without guiding marks. Note that the absorbed current signal shows some vertically running striations, due to a 50 *Hz* disturbance arising from the Swiss electric power net.

(i) The secondary-electron current arriving at the detector from Fe-islands (‘A’ domains) is notably *reduced* (by about a factor of 2) with respect to the secondary-electron current originating from the surrounding W-terraces. By the proximity rule, one would expect an *increase* of the transported current and indeed we observe an increase in the absorbed current in the SFM regime (see figure 4 in appendix A). But there the change at the Fe-W step is from one order of magnitude to two orders of magnitude smaller than the change in secondary-electron current. Thus, we learn from this observation that the secondary-electron yield on top of Fe is much smaller than the secondary-electron yield on top of W.
(ii) Secondary-electron imaging SFM detects a contrast within the terraces residing at the same vertical level: moving away from the steps towards the right-hand side, terraces are characterized by a narrow ‘dark’ stripe alongside the steps. These ‘B’-type domains have been highlighted in the enlarged image figure 3*d*. Further along towards, the right-hand side, terraces become brighter, rather abruptly, without any apparent ‘geometrical obstacle’ intervening, thus ruling out any proximity effect as the origin of the contrast of terraces at the same vertical level. ‘B’-type domains have been also green-dashed in the STM images figure 3*a*,*c* but are not apparent there as particular features. If we use the rule that the secondary-electron yield on top of Fe is smaller than the secondary-electron yield on top of W, we must conclude that this intra-terraces contrast, which is absent in STM imaging, proves the existence of a sizeable amount of Fe accumulating along W steps, which has gone undetected in the STM imaging. Notice that the right-hand side terraces towards the steps (some of these domains are marked with ‘C’ in figure 3*a*,*c*,*d*) are typically bright—i.e. they consist of W—and transform mostly into ‘B’ type domains upon going across the step. However, W-to-W transitions (‘C-to-C’ in figure 3*a*,*c*,*d*) also occur and appear as contrastless in the secondary-electron imaging SFM (figure 3*c*,*d*). These two facts allow us to conclude that, while in STM (and absorbed current SFM, see appendix A) the contrast is the result of the proximity rule, secondary-electron SFM distinguishes between Fe and W independently of their vertical position but by their different secondary-electron yield. This is an elemental specificity that distinguishes SFM from STM.

## 4. Discussion

### (a) Contrast

**Figure 9.**
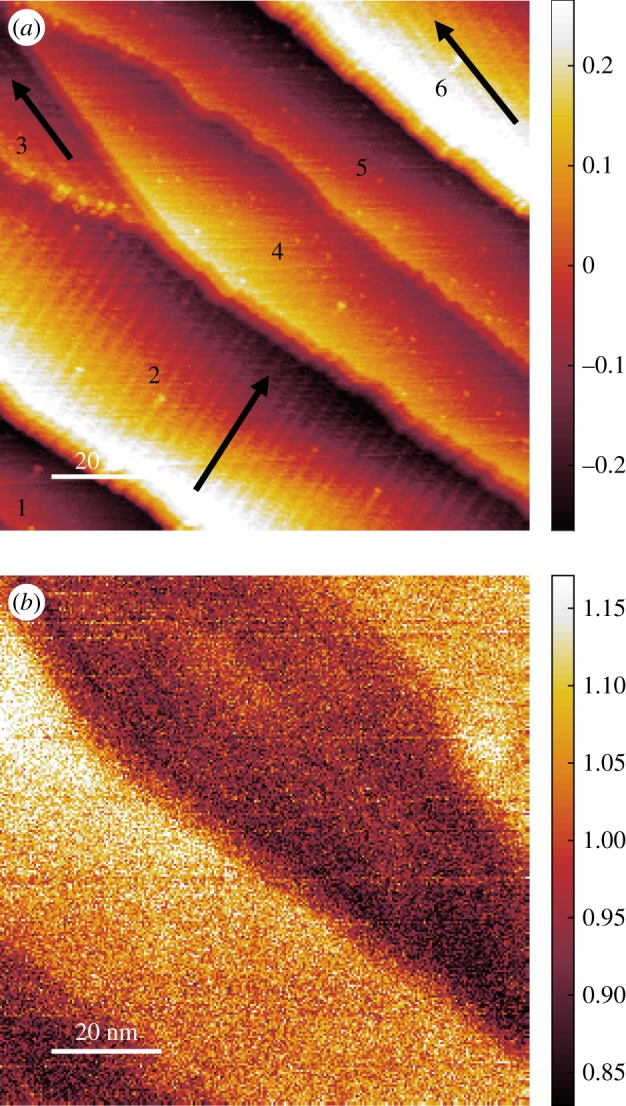
(*a*) STM image of W surface resulting from a standard cleaning procedure [12,13]. The terraces are numbered. A colour (vertical bar) is used to encode the tip displacement along *z*, in *nm*. A faint superstructure is visible on terraces ‘2’, ‘3’ and ‘6’, due to residual carbon impurities. Notice that the steps appear in pairs, slightly displaced with respect to each other—a ‘ghost’ phenomenon known to be produced by the tip ending with twin apices. (*b*) Secondary-electron image of the terraces given in (*a*). *V* =−29.8 volt, *d*=5±4 *nm* (estimate), average absorbed current *I*=125 *nA*. The contrast is between those terraces with superstructure and those without. The step dividing terraces ‘4’ and ‘5’ does not appear in the image as a separate feature.

**Figure 10.**
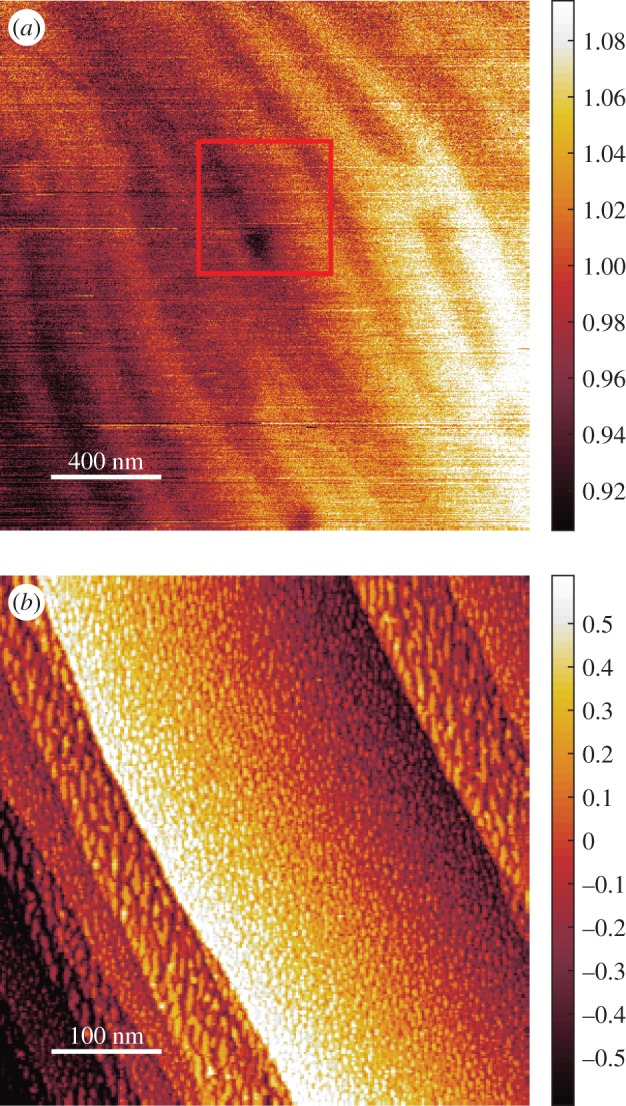
(*a*) Secondary-electron image of eight monolayers of Fe on W(110) (*V* = −46 *V*, *d*≈15 *nm*, *I* drifting from 10 to 50 *nA* while imaging). A colour code (vertical bar) is used to encode the secondary-electron intensity (normalized to the line average). The red square indicates the area which is investigated in (*b*) with STM. (*b*) STM image of the region marked by the red square in (*a*). A colour code (vertical bar) is used to encode the tip displacement in nm. The brighter terraces in (*a*) correspond to a finer structured Fe film in (*b*) (central terrace).

One defines the contrast between the images of objects *a* and *b* as the difference between the signals arising from *a* and *b* divided by their sum [2]. In STM, which is performed typically at constant current, one uses a relationship between constant-current and constant-height imaging [3], eqn. 8 to compute that the contrast across a single-atom step is some 10% (100% being the maximum figure attainable). This is the order of magnitude any other imaging technique must measure up to. In secondary-electron imaging SFM, we detect about a factor of two between the secondary-electron current from type ‘A’ Fe-domains with respect to the W surrounding, giving a figure of contrast of about 30%. A systematic quantitative analysis of the contrast in figure 3*b* is given in appendix B. Although we cannot exclude that other mechanisms intervene, like the interference of material waves excited in W but travelling through the Fe potential well before reaching the vacuum side [17],^4^ the most likely origin of the contrast is the material dependence of the secondary-electron yield [18,19]. Note, in fact, that the elemental sensitivity is not restricted to Fe and W: we also observe a strong contrast in secondary-electron generation also when going from ‘clean’ to carbon-covered W terraces (figure 9 in appendix C) and when going from Fe grown on ‘clean’ to Fe grown on carbon-covered W terraces (figure 10 in appendix C).

### (b) Spatial resolution

The vertical spatial resolution in secondary-electron imaging SFM is one atomic step (figure 3*b*). By comparing the horizontal size of type ‘A’ domains in figure 3*d* with the skeleton taken from figure 3*a*, it is apparent by visual inspection that these features have about the same size in both images. This fact and comparison of the ‘raw’ images in figure 2 show that the lateral spatial resolution obtained with SFM is somewhat lower than the resolution obtained by STM, but does not substantially deteriorate when going from STM (tip–target distance ≈0.2 *nm*) to secondary-electron imaging SFM (tip–target distance ≈7 *nm*). At a more quantitative level, we have determined the horizontal size of the steps separating type ‘A’ domains from the surrounding terrace with two different strategies. On the one hand, we have performed a line shape analysis of the boundary of various type ‘A’ domains and obtained an average step width of 1.1±0.2 *nm*. The step width observed in STM imaging (figure 3*c*) is marginally better than this figure. On the other hand, we have used a criterion based on the Fourier transform of images (see appendix B) to determine the wavelength necessary to describe the sharpest feature in the images (the boundaries of the type ‘A’ domains, the ‘C-to-C’ and ‘C-to-B’ boundaries). We observe a value between 0.8 and 1 *nm*, which agrees with the figure obtained by the line shape analysis. We consider these figures as an upper limit for the best horizontal spatial resolution inherent to the detection of one-atom-thick features with secondary-electrons SFM. Further data on how the horizontal spatial resolution in both secondary and absorbed current SFM depend on the tip-sample distance are given in appendix B.

## 5. Conclusion

We have observed a strong contrast in secondary-electron SFM imaging, originating from the material sensitivity of the secondary-electron yield. Our *table-top* set-up, which merges STM and SFM technology, allows highlighting complementary aspects of an image that would not be immediately accessible to a single technology alone. In this particular study, we have used this specific secondary-electron sensitivity to detect the Fe-atoms decorating the monoatomic W-steps which are not directly distinguishable in STM. One important added value of the SFM imaging mode is that electrons can escape the junction, so that they can be made available to further analysis, e.g. energy [10,11,20] and/or spin resolved spectroscopy [7,9]^5^ or for further processing. The astonishing high number of electrons actually escaping the junction^6^ speak also for the feasibility of imaging with sub-picosecond temporal resolution. The set of results presented here refer to a situation where the secondary electrons are excited and emerge from within a nanometre-sized spot placed just underneath the tip apex (which is itself at most some tens of nanometres far away), while the entrance of the secondary-electron detector is some centimetres far apart from the junction. Thus, a nanoscale quantum process, comprising field emission, secondary-electron production and electron transport in the presence of strong electric fields (of the order of 4 *V* *nm*^−1^), is shown to couple efficiently to a macroscopic environment, where electrons are detected using standard scanning electron microscopy methods (see pp. 235–236 of [12,13]).

## Figures and Tables

**Figure 5.**
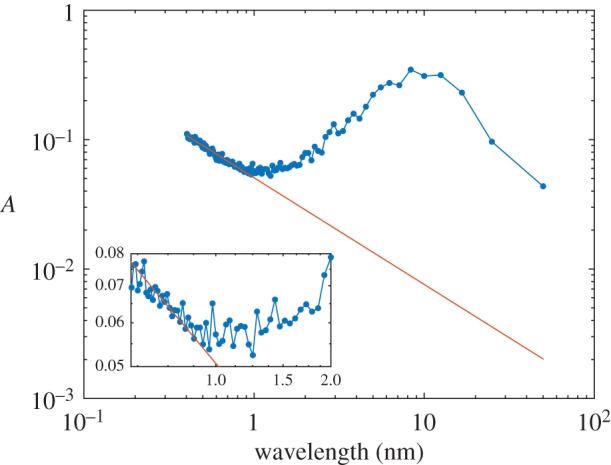
Amplitude *A* of the FFT of the image in figure 2*b*, averaged over the planar angular variable, as a function of the wavelength. The straight line is a fit to the statistical noise. Inset: zoom on the interval of wavelengths where the actual signal emerges from the statistical noise. (Online version in colour.)

**Figure 6.**
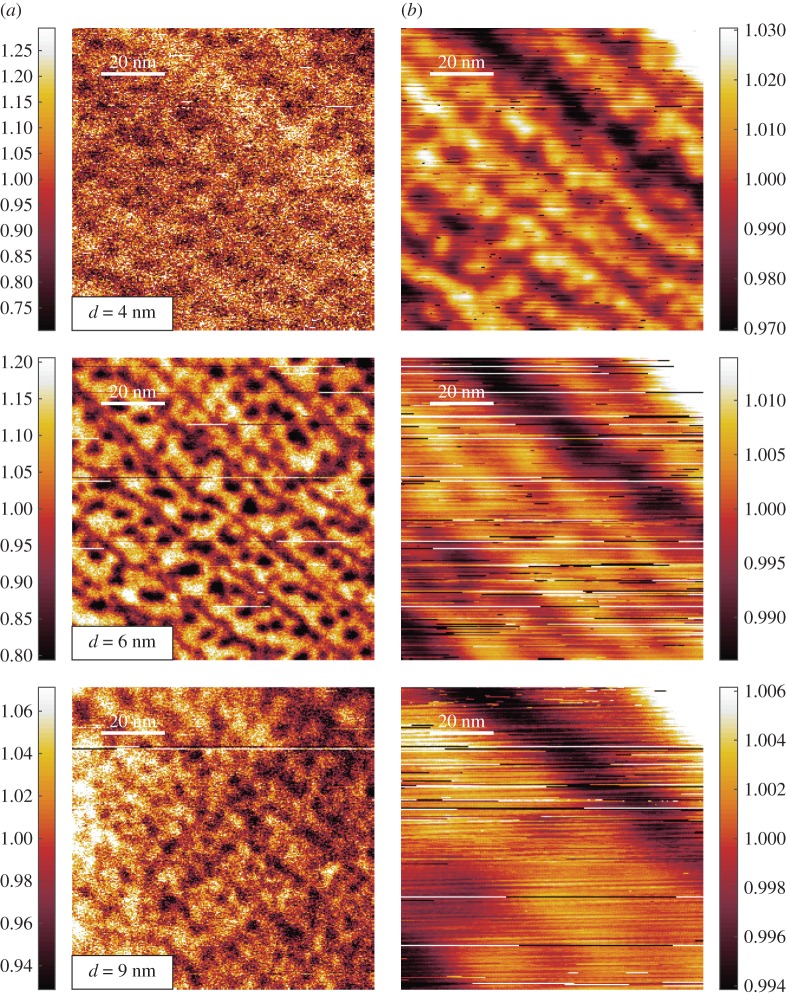
(*a*) Secondary-electron images of 0.2 atomic layers of Fe on W(110), taken at a fixed average current of about 400 nA for different distances (only selected images are shown). The colour code (vertical bar) is used to plot the ratio of the local secondary-electron intensity to its line average. The planar spatial scale is given by the white horizontal line. Note that, to image at variable *d* but keeping the current constant, the voltage must adapt [12,13]. (*b*) Absorbed current images taken simultaneously with the data on the left. The colour code (vertical bar) is used to plot the ratio of the local absorbed current to its line average. The planar spatial scale is given by the white horizontal line.

**Figure 7.**
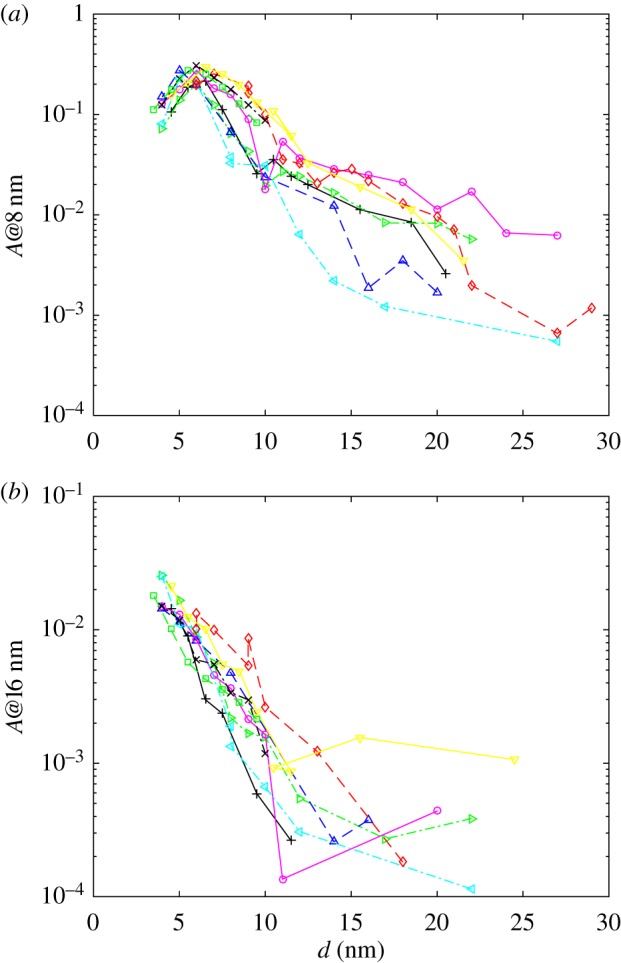
(*a*) Amplitude *A*@8 *nm* of the angle averaged Fourier transform of images of a 0.2 monolayers thick Fe film on W(110) at a wavelength of 8 *nm*, as a function of *d*. The maximum amplitude of the Fourier transforms is at 8±2 *nm*. For the compilation of this figure, images such as those shown in figure 6*a* are used. Various symbols and colours refer to a different set of data. (*b*) Amplitude *A*@16 *nm* of the angle averaged Fourier transform of current images in the regime of a 0.2 monolayers thick Fe film on W(110) at a wavelength of about 16 *nm*, as a function of *d*. For the compilation of this figure images such as those shown in figure 6*b* are used. Various symbols and colours refer to a different set of data.

**Figure 8.**
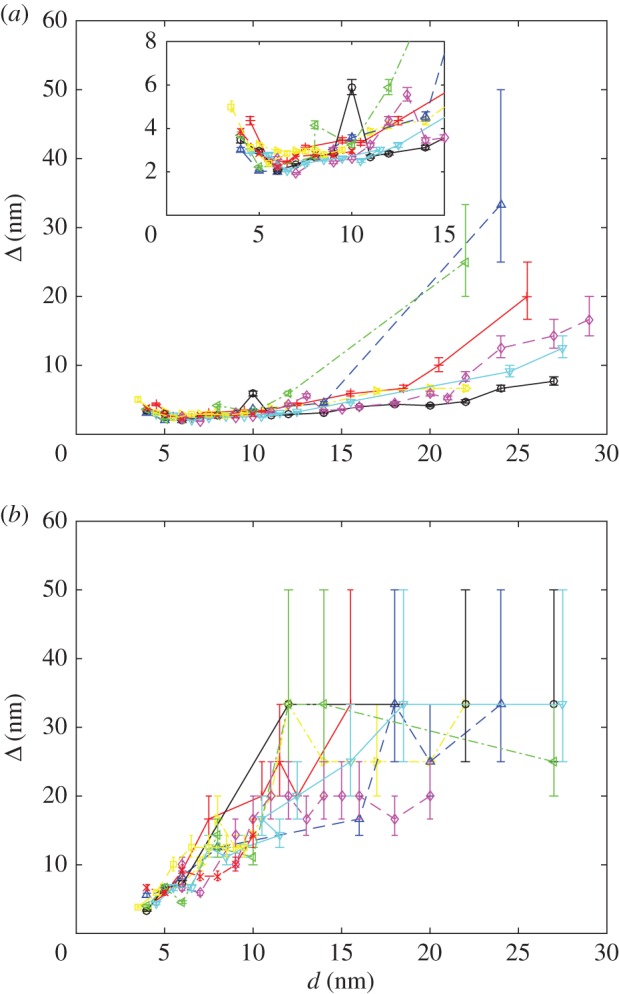
Threshold wavelength Δ, obtained with the algorithm illustrated in figure 5, as a function of *d* for (*a*) secondary-electron images such as those in figure 6*a* and (*b*) absorbed current images such as those of figure 6*b*. Various symbols and colours refer to a different set of data. The inset in (*a*) enhances the region *d*=4−15 *nm*. The error in *d* is about ±2 *nm*. The error in Δ refers to the uncertainty of establishing the threshold wavelength emerging from the noise in the Fourier spectrum of the images, and is determined by the value of the wavelength for the data point just before and just after the value picked up as emergence threshold.

## Footnotes

1 We use the definition of image contrast introduced by A. Michelson, see [2].

2 We call ‘secondary’ electrons both those which are elastically backscattered with the same energy as the primary beam and those which are produced at lower energies, i.e. those inelastically backscattered. In the present report, we do not analyse the energy of the detected electrons, but from preliminary experiments including energy analysis, we have indication that both types of electrons—elastic and inelastic backscattered—contribute to the secondary-electron image formation.

3 In principle, one could use the topography mapped via STM as a feedback for the lifted tip rather than a global plane. However, some drift during the STM/SFM operations and when moving from the STM to the SFM operation cannot be avoided (see §3b). Using a global plane, one, therefore, avoids the errors arising from referring to a topography deteriorated by the drift.

4 A scenario where the escaping of secondary electrons from the junction might be strongly dependent on the exact distance between the domain imaged and the tip apex—a kind of ‘proximity’ effect—can be excluded, as the contrast is seen within the very same terrace as well.

5 Preliminary experiments demonstrate that secondary electrons emitted with SFM are spin polarized at tip–target distances as small as 12 nm.

6 We find that about 1% (see also [12,13]) of the primary electron beam actually reaches the detector, the entrance of which covering a solid angle of about 0.2 sr. We are still puzzled by the large number of electrons actually escaping the junction in the actual experiment: the strong electric field existing at the position where electrons are produced systematically bends, in the simulations, the electron trajectory towards the surface. We are, however, sceptical about the capability of commercial trajectory simulations software in capturing the multiscale process described here [21].

## Data accessibility

Data are available from the ETH Zurich Data Archive: http://doi.org/10.5905/ethz-1007-66.

## Appendix A. Absorbed current imaging

To explain the transition from STM (figure 2*a*) to absorbed current SFM, we redraw figure 2*a* after the subtraction of a mathematical plane globally parallel to the scanned area (figure 4*a*). The schematic profile faced by the tip apex has now the sawtooth appearance sketched on top of figure 4*a*. The skeleton obtained in figure 2*a* is superimposed as continuous lines onto the image. The colour code used to render the displacement is given in the vertical bar. Accordingly, the tip has to perform a downward displacement when moving from the left-hand side to the right-hand side of a terrace, to keep a constant tunnel current. Thus, the right-hand side of a terrace is darker than the left-hand side, the brighter tone being recovered when going from left to right across a step. In this image, the ‘A’ domains (blue contour) always appear brighter than the surrounding terraces, as they reside on top of the terraces and the tip must be drawn upwards to keep the tunnel current constant. Notice the slight decrease of brightness within ‘A’ domains when moving from ‘southeast’ towards ‘northwest’. To explain this gradient, we point out that, strictly speaking, the sawtooth profile is not simply from ‘left to right’ (as simplified on top of figure 4*a*), but is running along the direction perpendicular to the overall step direction, i.e. from ‘southeast’ to ‘northwest’, producing the observed slight gradient of brightness. The building of the contrast in the STM figure 4*a* is explained by the proximity rule: the tunnel current into or out of regions of the target closer to the tip is larger (brighter colour).

Figure 4*b* shows the locally absorbed current during an SFM scan of the *same* area of figure 4*a*, at *d*=7±2 *nm*. The locally absorbed current is divided by the line average. The colour code used to render the normalized absorbed current is given in the vertical bar, darker tones corresponding to smaller relative currents. The skeleton obtained in figure 4*a* is superimposed as continuous lines onto the image (we have used the image figure 2*b*, taken simultaneously with figure 4*b*, for aligning the skeleton). The absorbed current image figure 4*b* can be explained on the basis of the sawtooth profile of figure 4*a*. The brightness decreases in going from the left hand-side of a terrace to the right-hand side, the brightness changing abruptly at the step-edges. The ‘A’ domains are the closest ones to the tip and appear brighter than the surrounding terrace. Thus, by comparing the STM figure 4*a* and the absorbed current figure 4*b* we learn that both images are built according to the same *‘proximity’ rule of thumb: regions residing close to the tip apex draw more current than regions residing farther away from it*, as expected from theoretical arguments [4,21]. However, despite this similarity, there are significant differences between the STM image figure 4*a* and the absorbed current image figure 4*b*. As evident from the values of the normalized current, the contrast in absorbed current SFM is only a few per cent, i.e. at least one order of magnitude smaller than the contrast in STM and secondary-electrons SFM. A systematic quantitative analysis of the contrast in absorbed current imaging is given in appendix B. Furthermore, the features of the image figure 4*b*—we refer mainly to the ‘A’ domains—appear significantly broader than in figure 4*a*, signalling that the horizontal spatial resolution is considerably worse in absorbed current imaging SFM. A systematic analysis of the horizontal spatial resolution in absorbed current imaging SFM is given in appendix B.

There is currently no model of electron production and scattering at such low energies, that, taking also into account the close proximity between tip and target, provides a quantitative explanation of the surprising good horizontal spatial resolution of secondary-electron SFM imaging (figure 2*b*) and its deterioration in absorbed current SFM (figure 4*b*). We point out, however, that the current absorbed by the target has two components: one originating from the primary beam—which should be spatially localized by the field emission process itself [22]—and one arising from the secondary electrons which leave the target but are turned back to it within short distances from where they have been excited [23] by the strong electric fields existing underneath the tip. Such ‘reabsorbed’ secondary electrons might extend to larger scales than the size of the primary electron beam [23], thus producing the ‘broadening’ of the features registered in current imaging. Thus, although there might be some practical advantages of current imaging at distances of 5–10 nm with respect to imaging at subnanometre distances (STM)—like being able to zoom over scan areas with much larger scanning range (the ‘zoom feature’ originally pointed out by Young himself [5,6])—absorbed current imaging SFM does not provide any new essential information with respect to direct tunnelling STM.

A technical detail is apparent from the comparison of figure 2*b* and the figure 4*b*: both absorbed current SFM (figure 4*b*) and secondary-electron SFM (figure 2*b*) detect a given feature at the same planar coordinate (if we set aside the small southeast shift due to the sawtooth phenomenon and the different broadening of the surface feature). This implies that the observed secondary electrons are originating from within the same spot where the current is arriving—a spot where supposedly strong electric fields prohibit secondary electrons from escaping [20]. This is a technical element of the physical process of imaging which is both experimentally compelling and essentially unexplained within current views of the nanoscale processes in the vicinity of this junction and their coupling to a macroscopic environment.

## Appendix B. Determination of contrast and horizontal resolution with Fourier transform

**(a) Algorithm**

To perform a systematic study of the amplitude of contrast and the horizontal spatial resolution of images, we have developed an algorithm based on the fast Fourier transform (FFT) [24] of images. The Fourier transform provides information about amplitudes and wavelengths of those Fourier components required to describe the recorded image. The amplitude *A* of the FFT is sought-after for contrast. Figure 5 plots the amplitude of the FFT of the image in figure 2*b*, averaged over the planar angular coordinate for improving the signal-to-noise ratio of the Fourier transform, as a function of the wavelength. The graph in figure 5 has a tail towards small wavelengths (less than 1 *nm*), representing the statistical noise component, like those features which result from sudden jumps of current. This tail is fitted in the graph by a continuous red line. The tail is followed by developing a sizeable Fourier component which describes the actual signal and undergoes a broad maximum at about *λ*=10 *nm*—the average horizontal size of the type ‘A’ domains in figure 2*b*. The amplitude at maximum is the maximum contrast attained in the image (about 30% in this instance). The threshold wavelength Δ at which the signal emerges from the noise is taken to be the wavelength required to describe the sharpest features in the image (between 0.8 and 1.0 *nm* in this instance, see the inset in figure 5).

**(b) Contrast: quantitative**

Figure 6 shows images in the secondary-electron SFM mode (*a*) and absorbed current mode SFM (*b*) of 0.20 monolayers of Fe on W(110), taken at different distances *d* with the same averaged absorbed current. More data for other values of *d* and at different average currents are available and are evaluated using the algorithm described above. The scales for the brightness are indicated in the corresponding vertical bars. The local currents (absorbed and secondary electron) have been divided by the line average, so that the contrast can be compared at glance. The build-up of the contrast follows the guidelines discussed in figures 2*b* and 4*b*. For several sets of such images, taken at different average currents and at different distances *d*, we have applied the algorithm illustrated in figure 5 in order to obtain information about the largest attainable image contrast and the threshold wavelength Δ. Figure 7*a* plots the amplitude *A*@8 *nm* of the Fourier transform of secondary-electron SFM images at 8 *nm* wavelength (the average horizontal size of the Fe ‘A’ domains in figure 6), which corresponds approximately to the maximum contrast attainable, as a function of *d*. In figure 7*b* *A*@16 *nm* of absorbed-current images SFM is plotted, as a function of *d*. The choice of a larger wavelength is due to the fact that the same features appear broader in current imaging than in secondary-electron imaging (see later the discussion about Δ), so that we need to go to larger wavelengths to pick up the true signal. The contrast of secondary-electron imaging is, in the *d* range 5–8 nm, of the order of 20% and at least one order of magnitude larger than the one in the current mode. This underlines the completely different origin of the contrast in the two imaging modes: a proximity mechanism in current images and a non-proximity one in secondary-electron imaging (see the discussion in the main text). Notice, however, that the graph *A*@8 *nm* versus *d* in the secondary electron mode reveals the existence of an optimum distance for maximum contrast, while the contrast of the current images increases monotonously towards smaller distances. The existence of an optimum distance might point to the fact that when *d* is too small, less secondary electrons succeed in escaping the junction and the contrast is suppressed by the detector noise. The decrease of the amplitude for higher values of *d*, instead, is probably due to the deteriorating spatial resolution (see later in figure 8), that averages the signal from high secondary-electron yield and low secondary-electron yield regions of the surface. The monotonous decrease of contrast in the current mode should be the result of both vertical sensitivity and horizontal resolution deteriorating with increasing distance, in agreement with models of field emission STM [4].

**(c) Horizontal resolution: quantitative**

Figure 8 reports the quantity Δ (introduced above) as a function of *d* for secondary-electron images (a) and current images (b). The Δ for secondary-electron imaging is, over the whole range of *d*, substantially smaller than the corresponding quantity in current imaging, both increasing with increasing distances (for large enough *d*). There is currently no model that correctly predicts the *d*-dependence of Δ or the remarkable difference in the two imaging modes. Note that, towards low distances, Δ(*d*) for secondary-electron imaging (inset in figure 8*a*) undergoes a minimum at about *d*=5−6 *nm*. This feature might be due to the deterioration of the signal at low distances (figure 6), so that it emerges from the noise at a larger threshold.

## Appendix C. Contrast mechanisms: elemental versus proximal

The contrast in secondary-electron images of Fe on W(110) in the submonolayer regime (figure 2*b*) points to a rule of thumb: ‘dark means Fe’ and ‘bright means W’. This elemental (chemical) specificity is supported by the images shown in figure 9. Figure 9*a*—an STM image of an area of a W(110)-surface—displays a number of terraces separated by monoatomic steps, running approximately along the diagonal. Terraces ‘2’, ‘3’ and ‘6’ have a clearly visible (albeit faint) fine structure, consisting of thin rows running along the direction indicated by the arrows. This fine structure is an indication that terraces are carbonized by an incomplete cleaning procedure which left some residual carbon impurities behind [14]. The contrast in the secondary-electron image figure 9*b* distinguishes precisely the carbonized terraces from the non-carbonized ones, while the existence of a monoatomic step dividing terraces ‘4’ and ‘5’ is not registered by the secondary-electron image. This allows to exclude a proximity effect for the formation of contrast in secondary electron imaging and strongly points to the elemental origin of the contrast. Of course, the presence of carbon also produces the particular, raw-like atomic structure seen in figure 9*a*. The atomic structure itself could also contribute to the specific secondary-electron yield observed on carbonized surfaces, thus adding a further source of contrast (a morphological one) in addition to the elemental one.

Figure 10*a* shows a secondary-electron image of an eight-monolayers Fe film grown on W(110). The contrast in the brightness cannot be a Fe–W contrast, as the W is completely buried beneath the Fe film. However, the STM image (figure 10*b*) of the region marked by a red square window in figure 10*a* reveals that the origin of the selective secondary-electron production is the actual Fe morphology, the brighter regions corresponding to a finer structured Fe films (middle terrace) that typically grows on carbonized terraces, as opposed to the platelets-like morphology of Fe films grown on ‘clean’ W surfaces (the terraces on the left and on the right of the central one) [14]. It appears that the secondary-electron production is also very sensitive to the morphology of the films on the atomic level.
